# The Circulation of Type F *Clostridium perfringens* among Humans, Sewage, and *Ruditapes philippinarum* (Asari Clams)

**DOI:** 10.3390/pathogens9080669

**Published:** 2020-08-18

**Authors:** Keita Yanagimoto, Kosei Uematsu, Takaya Yamagami, Eiji Haramoto

**Affiliations:** 1 Department of Microbiology, Yamanashi Institute of Public Health and Environment, 1-7-31 Fujimi, Kofu, Yamanashi 400-0027, Japan; yanagimoto-amvs@pref.yamanashi.lg.jp (K.Y.); uematsu-syy@pref.yamanashi.lg.jp (K.U.); yamagami-yjz@pref.yamanashi.lg.jp (T.Y.); 2 Environmental and Social System Science Course, University of Yamanashi, 4-3-11 Takeda, Kofu, Yamanashi 400-8511, Japan; 3 Interdisciplinary Center for River Basin Environment, University of Yamanashi, 4-3-11 Takeda, Kofu, Yamanashi 400-8511, Japan

**Keywords:** asari clam, *cpe*-positive *C*. *perfringens*, *Ruditapes philippinarum*, multilocus sequence typing, wastewater treatment plant

## Abstract

*Clostridium perfringens* is an important pathogen that is responsible for gastroenteritis; the causative agent for the symptoms is *C. perfringens* enterotoxin (CPE), which is mainly produced by type F *C. perfringens*. Since shellfishes may gather *C. perfringens* in the water environment, this study estimated the potential circulation of type F *C. perfringens* among humans, sewage, and *Ruditapes philippinarum* (asari clams) as a result of sewage pollution. A comparison of the characteristics among the isolates from 86 sewage influents, 36 effluents, 76 asari clams, and 37 humans was conducted. Serotyping, *cpe* genotyping, and toxin genotyping showed that *C*. *perfringens* with a plasmid IS*1151* sequence downstream of *cpe* was predominant among sewage influents, effluents, humans, and asari clams. Multilocus sequence typing suggested that some isolates from a human, sewage influents, effluents, and asari clams were linked to each other. These results demonstrated that asari clams are the necessary infection sources of *C. perfringens* responsible for carriers and foodborne diseases, and that these pathogens from humans infected by asari clams can pollute the water environment. It is useful to assess bacteria such as *C. perfringens* isolates from sewage to estimate the trend of those from the community.

## 1. Introduction

*Clostridium perfringens*, a spore-forming, Gram-positive, rod-shaped, anaerobic bacterium, may induce gastroenteritis with diarrhea and abdominal pain. Categorizing *C. perfringens* is based on toxin production (alpha, beta, epsilon, and iota toxins) divided into types A–E [1]. Recently, the new categorization of *C. perfringens* that produces alpha toxin and *C. perfringens* enterotoxin (CPE) is type F, and strains that possess alpha and NetB toxins are now categorized as type G [1]. CPE is mainly responsible for gastrointestinal symptoms, and importantly, in Japan, an average of 20–40 *C. perfringens*-associated foodborne outbreaks (involving up to 3000 victims) were recognized each year, which is the second leading cause of bacterial food poisoning in the country [2]. *Clostridium perfringens* producing CPE and/or CPE in feces from patients has been found in outbreaks [3,4,5]. CPE-producing *C. perfringens* causes both foodborne outbreaks and non-foodborne diarrhea [6,7,8] and was isolated from healthy humans, animals with enteric diseases, retail foods, and environmental samples [9,10,11,12,13,14,15,16,17,18,19,20,21].

*Clostridium perfringens* is an effective human sewage indicator because of its direct link and environmental stability [22,23]. Additionally, type F *C. perfringens* was reported as an essential indicator of human fecal pollution in aquatic environments because most of the isolates were not observed in livestock-related samples including animal feces and wastewater of a pig farm and an abattoir but observed in human feces and sewage influents and effluents [18]. Various studies have shown that shellfishes were contaminated with viral pathogens and induced gastroenteritis [24,25,26,27,28] due to the greater concentrations of pathogen accumulation in sewage than those of the water surrounding them [29,30,31,32,33]. Therefore, type F *C. perfringens* discharged in sewage effluents should contaminate seawater and may be accumulated by shellfishes. If this is true, the impact of shellfishes on type F *C. perfringens* infection to humans and infected humans on the water environment are critical; however, these are not well known.

This study estimated the importance of *Ruditapes philippinarum* (asari clams) as an infection source and humans as a pollution source of type F *C. perfringens* by comparing the characteristics among the isolates from influents and effluents of two wastewater treatment plants (WWTPs), asari clams bought in the same study areas and humans.

## 2. Results

### 2.1. Distribution of Type F C. perfringens

**Figure 1:**
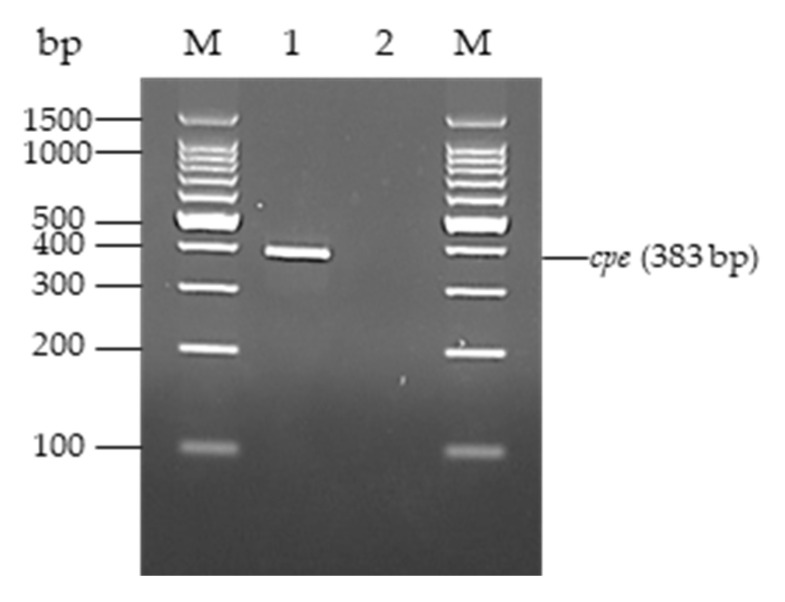
PCR assay identifying *cpe* gene of *C. perfringens* isolates using 2.5% agarose gel electrophoresis. M: 100 bp DNA Ladder (Takara Bio, Kusatsu, Japan); 1: *cpe*-positive *C. perfringens*; 2: *cpe*-negative *C. perfringens.*

**Figure 2:**
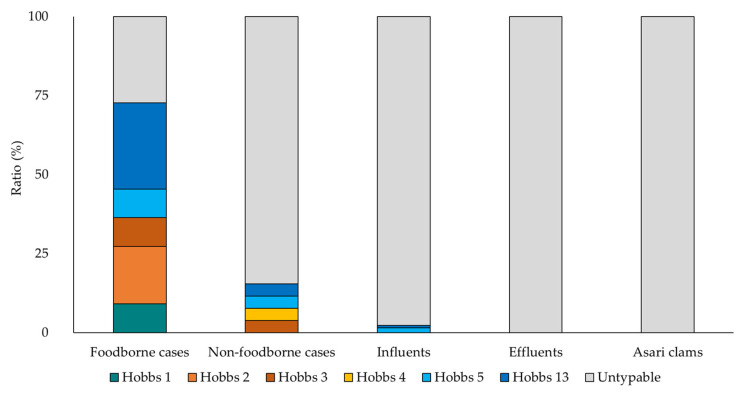
Characterization of the isolates from foodborne cases, non-foodborne cases, influents, effluents, and asari clams by serotyping.

**Figure 3:**
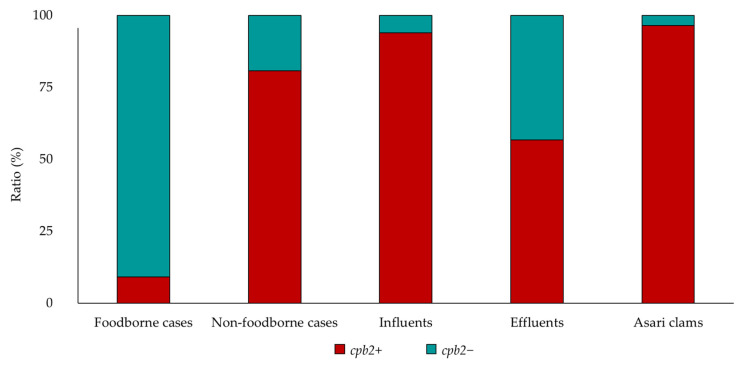
Characterization of the isolates from foodborne cases, non-foodborne cases, influents, effluents, and asari clams positive or negative for *cpb2*.

**Figure 4:**
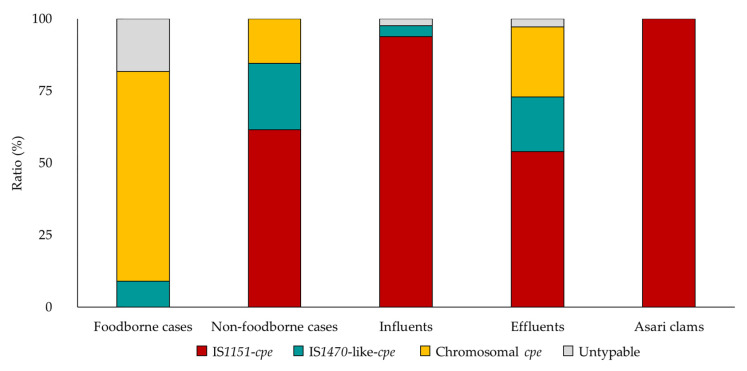
Characterization of the isolates from foodborne cases, non-foodborne cases, influents, effluents, and asari clams by *cpe* genotyping assay.

Using conventional PCR in this study, the isolation rates of *cpe*-positive *C. perfringens* from sewage influent, effluent, and asari clams including shells were 80% (69/86), 56% (20/36), and 9% (7/76), and 131, 37, and 28 isolates were obtained, respectively (Figure 1). Among the isolates from foodborne cases, 73% (8/11) were typable serotypes, whereas 98% (128/131), 100% (37/37), 100% (28/28), and 85% (22/26) of the isolates from influents, effluents, asari clams, and non-foodborne cases were untypable, respectively (Figure 2). All isolates such as 37 human isolates were categorized as *C. perfringens* type F, which is positive for *C. perfringens* alpha toxin gene and *cpe*. Additionally, 94% (123/131), 57% (21/37), 96% (27/28), and 81% (21/26) of the isolates from influents, effluents, asari clams, and non-foodborne cases, respectively, were positive for *C. perfringens* beta2 toxin gene (*cpb*2). Conversely, only one (9%) isolate from a foodborne case had *cpb2* (Figure 3). The ratio(s) of the isolates with a chromosomal *cpe* among those from foodborne cases was 73% (8/11), while those with a plasmid IS*1151* sequence downstream of *cpe* among those from influents, effluents, asari clams, and non-foodborne cases were 94% (123/131), 54% (20/37), 100% (28/28), and 62% (16/26), respectively (Figure 4). The ratio of the isolates with a plasmid IS*1151*-*cpe* among the isolates from influents was significantly higher than those from effluents (*p* < 0.01). In total, the ratio of the isolates with *cpb*2 among the isolates with a plasmid IS*1151*-*cpe* (96%) was significantly higher than those with a chromosomal *cpe* (14%) (*p* < 0.01). The isolates with a plasmid IS*1470*-like sequence downstream of *cpe* were detected from all sources but asari clams. Table 1 and Table 2 show the number of isolates and isolation period from influents and effluents, respectively. No seasonal trend for the isolation from each kind of sample was observed and no significant difference between WWTP-A and WWTP-B for the isolation was also observed.

### 2.2. Characterization of Type F C. perfringens by Multilocus Sequence Typing (MLST)

**Figure 5:**
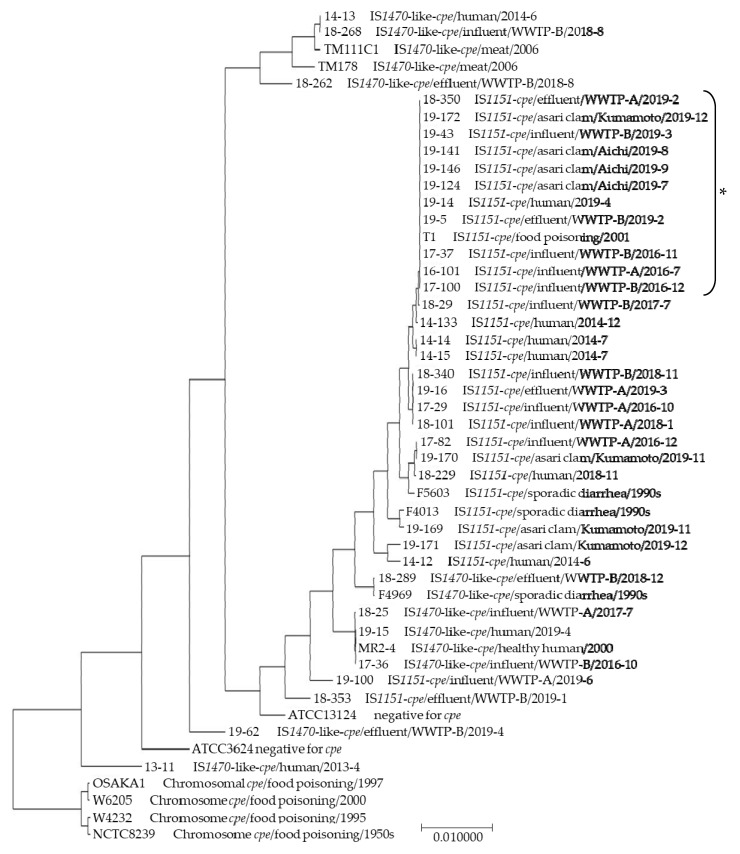
Genetic characterization of 36 type F *C. perfringens* isolates and 13 collection strains by multilocus sequence typing (MLST). Construction of phylogenetic tree based on a concatenated sequence of eight housekeeping genes was conducted with the Clustal W format. The asterisk (*) shows asari clam clones. A variation scale is supplied at the bottom.

The genetic comparison of the isolates by MLST assessing eight housekeeping genes has shown that *C*. *perfringens* with a plasmid IS*1151*-*cpe* or a plasmid IS*1470*-like-*cpe* were divided into two clusters, regardless of their sources, a cluster only comprising isolates with a IS*1470*-like-*cpe* and a cluster mainly comprising those with a plasmid IS*1151*-*cpe*, both of which were different from strains with a chromosomal *cpe*. In a cluster that principally comprises the isolates with a plasmid IS*1151*-*cpe*, isolates from a human, four sewage influents, two effluents, and four asari clams have a completely conserved sequence of eight genes identified as the asari clam clone. The ratios of asari clam clone were 40% (4/10), 50% (2/4), and 57% (4/7) among the tested isolates with a plasmid IS*1151*-*cpe* from influents, effluents, and asari clams, respectively. Strain T1 isolated from a foodborne case described by Tanaka et al. [34] was also categorized as an asari clam clone but strains TM111C1 and TM178 isolated from retail meats in Japan [15] were not classified (Figure 5). These clones were isolated from July to December 2016 or from February to December 2019.

## 3. Discussion

This study has shown the characteristics of type F *C. perfringens* isolated from sewage influents, effluents, asari clams, and humans to check whether they are predominant from those of humans and environments in the study area. Additionally, we have shown the genetic relationship of the isolates to show their behavior in the water environment, as well as the isolation rate of the isolates in asari clams to consider them as an infection source for healthy humans and foodborne cases.

We explored the characteristics of the isolates from influents, effluents, asari clams, and humans to understand the behavior of type F *C. perfringens* due to four reasons. First, asari clams can probably show an infection source of type F *C. perfringens* for humans because of the accumulation of pathogens in the water environment [29,30,31,32,33]. Second, it is impossible to know the prevalence of type F *C. perfringens* isolated from clinical and nonclinical cases using the present passive surveillance system because this surveillance system hardly reports nonclinical cases to public health officials [35]. Third, the characteristics of *Salmonella enterica* isolated from sewage influents and humans were closely associated with each other, indicating that isolates from unreported cases were obtained from influents in our previous study [36]. Finally, the isolates from humans infected by asari clams may become a pollution source of water environment via sewage influents and effluents when the impact of asari clams on human infection is significant. The isolation rates of type F *C. perfringens* were 80%, 56%, and 9% from influents, effluents, and asari clams, respectively, which were higher than the previous report of 29% and 32% from influents and effluents, respectively [18]. The differences in isolation rates may be because of the difference in the amount of samples to be cultured in an enrichment broth and that in the geographical circumstances.

The results of serotyping, toxin genotyping, and *cpe* genotyping assay indicated a different distribution between sewage influents, effluents, humans in non-foodborne cases, and asari clams and humans in foodborne cases. The isolate with an untypable serotype, positive for *cpb*2, a plasmid IS*1151*-*cpe*, was predominant in the isolates from influents, effluents, humans in non-foodborne cases, and asari clams, while that with a typable serotype, negative for *cpb*2, and a chromosomal *cpe* was predominant in the isolates from humans in foodborne cases. Most of the type F *C. perfringens* isolated from influents are probably not from foodborne cases but from non-foodborne cases, such as healthy humans, as observed in our results and in the following three facts. First, several reports have shown that the frequency of type F *C. perfringens* among healthy humans was 6–31% [9,10,11,12]. Second, the predominant *cpe* locus of the isolates from healthy humans and sporadic diarrhea cases in Japan was a plasmid with a IS*1151* sequence [37,38]. Finally, type F *C. perfringens* was isolated from few human-unrelated samples [18]. These results showed that the isolates from sewage influents have a great association with those from healthy humans. Deguchi et al. have shown that 11 strains with a chromosomal *cpe*, which were all tested in their study, were negative for *cpb*2, whereas some of those with a plasmid *cpe* were positive [39]. Our results show that the ratio of the isolates with *cpb*2 among the isolates with a plasmid IS*1151*-*cpe* was significantly higher than that with a chromosomal *cpe* and the study by Deguchi et al. [39] demonstrated that the possession of *cpb*2 is associated with the locus of *cpe*.

Analysis by MLST indicated that the isolates with plasmid IS*1151*-*cpe* from a human, four influents, two effluents, and four asari clams were categorized as an identical clone identified as the asari clam clone, which was clearly different from strains with chromosomal *cpe* and plasmid IS*1470*-like-*cpe* isolated from meats. In addition, this result agrees with studies that have shown that strains with chromosomal *cpe* belonged to a single distinct cluster [39,40] and exhibited different genetic characteristics from those with plasmidal *cpe* revealed by analysis based on whole genome sequencing [41,42]. Additionally, the asari clam clone was predominant among the isolates from influents (40%), suggesting that the asari clam clone is also predominant among unreported human isolates, effluents (50%), and asari clams (57%) with plasmid IS*1151*-*cpe* tested in this study. These results suggested that asari clams have an important impact on infection of type F *C. perfringens* to humans and that the asari clam clones pollute the water environment and are widely distributed among humans and water environment through asari clams. Although type F *C. perfringens* may be circulating among humans, water environment, and bivalves, it is difficult to experimentally validate this hypothesis, lacking the result of human isolates in Aichi or Kumamoto. Additionally, performing whole genome sequencing would need to prove this hypothesis as a method for genetic comparison.

The frequency of type F *C. perfringens* isolation among healthy humans (6–31%) [9,10,11,12] is much higher than that of *Salmonella* spp. (0.013%) [43], enterohemorrhagic *Escherichia coli* (EHEC) (0.2%) [44], and *Campylobacter* spp. (1%) [45], which are the principal pathogens of gastroenteritis in Japan [2]. This means that type F *C. perfringens* is more often detected from infection sources such as foods and/or environments. The isolation rate of type F *C. perfringens* vegetative cells and spores from investigated foods, principally meat products (0–5%) [11,14,15,16,46,47,48], was lower than that of *Salmonella* (8–10%), EHEC (16.6%), and *Campylobacter* (21–27%) from foods [49,50,51,52]. However, the actual infection risks of type F *C. perfringens* infection from foods would be higher since most of these contaminated foods are heated well before eating them, so bacteria in foods would be killed excluding type F *C. perfringens* spores, which are heat resistant. In our study, the isolation rate of type F *C. perfringens* from asari clams heated at 80 °C for 10 min was 9%, which is the same with the results of previous studies showing isolation rates of 8% and 12%, respectively, in scallops and oysters [16,19]. Zhang et al. [53] described that *C. perfringens* in beach sand showed a slower decay than that in seawater, supporting our results that showed a high isolation rate of asari clams in beach sand, while those of benthic crabs in a sewage-polluted estuary and fish were 1% and 1.4%, respectively [54,55]. These results suggested that the isolation rate of type F *C. perfringens* spores from bivalves is higher than those from other foods, primarily meat products. Additionally, probably the high detection frequency of type F *C. perfringens* from healthy humans is partially because of bivalves such as asari clams since our MLST result has shown that the sequences of the isolates from a human and asari clams were linked to each other, but those from retail meats were clearly different. The MLST result of Matsuda et al. has shown that some of the isolates from humans were ST41 [38] by Xiao’s scheme [40], which is the same as the asari clam clone.

Foodborne outbreaks due to *C. perfringens* are principally responsible for those with a chromosomal *cpe* since they have spores that are resistant to heat (100 °C for 10 min), cold, osmotic stress, and nitrites, whereas those spores with a plasmid *cpe* are relatively sensitive to these [34,56,57,58,59,60]. In our study, there was no isolate with a chromosomal *cpe* from asari clams, in which the isolates with a plasmid IS*1151*-*cpe* were identified. Regardless of this, here, we believe that type F *C. perfringens* in bivalves such as asari clams induce both asymptomatic carriers and foodborne outbreaks because of the following three reasons. First, several reports have argued that foodborne outbreaks are caused or associated by heat-sensitive *C. perfringens* with a plasmid IS*1151*-*cpe* or IS*1470*-like-*cpe* [11,34,57,60,61], one of which includes strain T1 observed to belong to asari clam clones in our study. Additionally, Kiu et al. have reported that *C. perfringens* strains with a plasmid IS*1151*-*cpe* or IS*1470*-like-*cpe* were predominantly associated with food poisoning cases [41]. Moreover, Grant et al. suggested that the isolate with a plasmid *cpe* formed spores with high resistance to heating at 95 °C for 30 min [62]. Second, the isolates with a chromosomal *cpe* were recognized from effluents, meaning there may be an accumulation of those by bivalves. Finally, there may be some foods that are heated below 100 °C for 10 min in accordance with the fact that the heating condition at 80 °C for 10 min, at which the spores of the isolates with a plasmid *cpe* can survive, could open all asari clams in our study. Thus, the foods including bivalves, such as clam chowder or seafood curry, can cause food poisoning by type F *C. perfringens* unless the foods are served right after cooking since the requirement for food to still be consumed without reaching a toxin level is less than 10^5^ organisms/g [63].

The ratio of the isolates with a plasmid IS*1151*-*cpe* among the isolates from influents was 94%, which was significantly higher than that from effluents (54%) in which the relatively high proportion of the isolates with chromosomal *cpe* (24%) was observed. The proportional modification of F-specific RNA coliphage genogroups via a wastewater treatment process was described previously [64]. Similarly, it is plausible that the different behaviors of the isolates through a wastewater treatment process depend on their locus of *cpe* or other factors. Considering spore formation, the isolates with a chromosomal *cpe* are more resistant to some kinds of stresses than those with a plasmid *cpe* and other *C. perfringens* [34,57,58,59,60,61], raising the possibility that the isolates with a chromosomal *cpe* are resistant to some stress in WWTPs, which needs further studies to be experimentally proven.

## 4. Materials and Methods

### 4.1. Sample Collection

Sewage influent and effluent samples were collected from two WWTPs (WWTP-A and WWTP-B) located in Yamanashi, Japan, monthly for 43 months between July 2016 and January 2020 and for 18 months between August 2018 and January 2020, respectively. These WWTPs serve a population of ~350,000 and treat a total of ~180,000 m^3^/day of wastewater. All samples were collected in normal weather and stored at 4 °C after sampling and tested within 24 h. Seventy-six packs of asari clams collected in Aichi or Kumamoto, central and southern parts of Japan, ~100 and ~750 km apart from estuary downstream of the effluents, respectively, were bought from supermarkets in Yamanashi between June 2019 and January 2020.

### 4.2. Isolation of Clostridium Perfringens

Four hundred milliliters of each sewage sample was centrifuged at 21,000× *g* for 25 min to obtain 2-mL suspensions. Subsequently, 0.1 mL of each suspension and 10 asari clams from each pack were added to 10 and 250 mL of thioglycolate broth (Nissui Pharmaceutical, Tokyo, Japan), respectively, followed by heating at 80 °C for 10 min and incubation at 35 °C for 20–24 h. Asari clams with shells were examined in this study because they are usually cooked in foods together, such as miso soup in Japan. The culture was inoculated onto CW agar plates (Nissui Pharmaceutical) that contained 50% egg yolk-enriched saline (Kyokuto Tokyo, Japan) and then incubated under anaerobic conditions at 35 °C for 20–24 h. Ten suspected colonies showing lecithinase production and lactose fermentation were isolated and suspended in sterilized distilled water, and DNA was extracted by heating at 100 °C for 10 min. The detection of *cpe* of the isolates was performed by conventional PCR using Thermal Cycler Dice Touch TP350 (Takara Bio). The amplification of *cpe* was performed with a primer pair as listed in Table 3. Each 25-μL reaction mixture contained 2.5 μL of template DNA, 12.5 μL of 2× Multiplex PCR Buffer, 0.125 μL of 2× Multiplex PCR Enzyme Mix (Takara Bio), and 2 μL each of 2.5-pmol/μL forward and reverse primers. PCR was performed under the following conditions: Initial denaturation at 94 °C for 1 min, followed by 35 cycles at 94 °C for 5 s, and 64 °C for 45 s. PCR products were electrophoresed on a 2.5% agarose gel with ethidium bromide, and the isolates with PCR products of 383 bp were found to be *cpe*-positive *C*. *perfringens*.

### 4.3. Human Isolates

Eleven isolates from human feces in foodborne cases and 26 isolates from those in non-foodborne cases in Yamanashi, which were obtained from administrative inspections by the Yamanashi Institute of Public Health and Environment between 2012 and 2019, were used. One isolate was used from each case.

### 4.4. Characterization of the Isolates

*C. perfringens* isolates with *cpe* were distinguished by serotyping, *cpe* genotyping assay, and toxin genotyping. Serotyping was performed using commercial *Clostridium perfringens* type A antisera set (Denka Seiken, Tokyo, Japan), and *cpe* genotyping assay was performed using the multiplex PCR described by Miyamoto et al. [65] to know the locus of *cpe*, including chromosomal *cpe* and plasmid *cpe* (IS*1151* or IS*1470*-like sequence downstream of *cpe*). Toxin genotyping was performed using the multiplex PCR described by van Asten et al. [66].

### 4.5. Statistical Analysis

Differences between the rate of *C. perfringens* isolates with *cpb2* among the isolates with a plasmid IS*1151*-*cpe* and those with a chromosomal *cpe* and the rate of *C. perfringens* isolates with a plasmid IS*1151*-*cpe* from influents and those from effluents was compared using the chi-square test. Moreover, *p* < 0.01 was considered statistically significant.

### 4.6. MLST Analysis

The representative 36 isolates listed in Table 4 were assessed using the MLST comparing sequence of concatenated eight housekeeping genes, such as *plc*, *colA* (toxin genes), *nadA*, *pgk* (putative metabolic genes), *sodA*, *groEL* (stress response gene), *sigK* (sigma factor involved in sporulation), and *gyrB* (DNA replication gene) to reveal the relationship among the isolates [39]. Additionally, sequence data of these genes obtained from GenBank (accession numbers AB477535, AB477536, AB477540–AB477542, AB477558, AB477568, AB477575, AB477579, AB477582, AB477585, AB477586, AB477588–AB477590, AB477594–AB477596, AB477612, AB477622, AB477629, AB477633, AB477636, AB477639, AB477640, AB477642–AB477644, AB477648–AB477650, AB477666, AB477676, AB477683, AB477687, AB477690, AB477693, AB477694, AB477696–AB477698, AB477702–AB477704, AB477720, AB477730, AB477737, AB477741, AB477744, AB477747, AB477748, AB477750–AB477752, AB477756–AB477758, AB477774, AB477784, AB477791, AB477795, AB477798, AB477801, AB477802, AB477804–AB477806, AB477810–AB477812, AB477828, AB4778838, AB477845, AB477849, AB477852, AB477855, AB477856, AB477858–AB477860, AB477864–AB477866, AB477882, AB477892, AB477899, AB477903, AB477906, AB477909, AB477910, AB477912–AB477914, AB477918–AB477920, AB477936, AB477946, AB477953, AB477957, AB477960, AB477963, AB477964, and AB477966for strains ATCC3624, ATCC13124, F4013, F4969, F5603, MR2-4, NCTC8239, OSAKA1, T1, TM111C1 TM178, W4232, and W6205) were also assessed. Sequence data were analyzed with the Clustal W format using a genetic information processing software (GENETYX Ver. 13, GENETYX, Tokyo, Japan).

### 4.7. Nucleotide Sequence Accession Numbers

The sequence data obtained in this study were deposited in the DNA Data Bank of Japan with accession numbers LC548776 to LC549063.

## 5. Conclusions

In conclusion, our results suggest that asari clams are an important infection source of type F *C. perfringens* to cause asymptomatic carriers and foodborne diseases. In addition, the isolates from humans infected by asari clams should become a pollution source of water environment through sewage influents and effluents and be widely distributed among the human and water environment. Sewage samples would provide several hygiene information about not only a specific human but also all the humans in the area, contributing to the understanding of what kinds of enteric pathogens spread in the community and investigating whether suspicious foods are infection sources. When the dynamics of pathogens are explored, it would be efficient to assess the isolates from sewage, reflecting the trend of those from the community.

## Figures and Tables

**Table 1:** Isolation of type F *C. perfringens* from sewage influents of wastewater treatment plants (WWTP)-A and WWTP-B.

Period		*cpe* Genotyping Assay							
		Chromosomal *cpe*		IS*1151*-*cpe*		IS*1470*-Like-*cpe*		Untypable *cpe*	
		A	B	A	B	A	B	A	B
2016	7	0	0	2	3	0	0	0	0
	8	0	0	1	1	0	0	0	0
	9	0	0	1	1	0	0	0	0
	10	0	0	3	0	0	1	0	0
	11	0	0	1	1	0	0	0	0
	12	0	0	2	4	0	0	0	1
2017	1	0	0	3	3	0	0	0	0
	2	0	0	2	2	0	0	0	0
	3	0	0	5	2	0	0	0	1
	4	0	0	3	3	0	0	0	0
	5	0	0	0	1	0	0	0	0
	6	0	0	0	1	0	0	0	0
	7	0	0	1	1	2	0	0	0
	8	0	0	1	2	0	0	0	0
	9	0	0	0	3	0	0	1	0
	10	0	0	2	3	0	0	0	0
	11	0	0	1	2	0	0	0	0
	12	0	0	0	3	0	0	0	0
2018	1	0	0	2	3	0	0	0	0
	2	0	0	1	0	0	0	0	0
	3	0	0	1	1	0	0	0	0
	4	0	0	1	2	0	0	0	0
	5	0	0	2	0	0	0	0	0
	6	0	0	0	1	0	0	0	0
	7	0	0	0	1	0	0	0	0
	8	0	0	1	0	0	1	0	0
	9	0	0	1	3	0	0	0	0
	10	0	0	1	4	0	0	0	0
	11	0	0	1	1	0	0	0	0
	12	0	0	2	3	0	0	0	0
2019	1	0	0	0	1	0	0	0	0
	2	0	0	0	1	0	0	0	0
	3	0	0	1	2	0	0	0	0
	4	0	0	1	0	0	0	0	0
	5	0	0	2	0	0	0	0	0
	6	0	0	4	0	0	0	0	0
	7	0	0	2	2	0	0	0	0
	8	0	0	3	0	0	0	0	0
	9	0	0	2	0	0	0	0	0
	10	0	0	1	0	0	0	0	0
	11	0	0	0	0	1	0	0	0
	12	0	0	3	1	0	0	0	0
2020	1	0	0	0	1	0	0	0	0
Total		0	0	60	63	3	2	1	2

A shows WWTP-A; B shows WWTP-B.

**Table 2:** Isolation of type F *C. perfringens* from sewage effluents of WWTP-A and WWTP-B.

Period		*cpe* Genotyping Assay							
		Chromosomal *cpe*		IS*1151*-*cpe*		IS*1470*-Like-*cpe*		Untypable *cpe*	
		A	B	A	B	A	B	A	B
2018	8	0	0	0	0	0	2	0	0
	9	5	0	0	0	0	0	0	0
	10	0	0	0	0	0	0	0	0
	11	0	0	1	0	1	0	0	0
	12	0	0	1	0	0	2	0	0
2019	1	0	0	0	1	0	1	1	0
	2	0	0	3	1	0	0	0	0
	3	0	0	1	1	0	0	0	0
	4	0	0	0	0	0	1	0	0
	5	0	0	0	1	0	0	0	0
	6	0	0	0	0	0	0	0	0
	7	0	1	6	1	0	0	0	0
	8	0	2	1	0	0	0	0	0
	9	0	1	0	0	0	0	0	0
	10	0	0	0	1	0	0	0	0
	11	0	0	0	1	0	0	0	0
	12	0	0	0	0	0	0	0	0
2020	1	0	0	0	0	0	0	0	0
Total		5	4	13	7	1	6	1	0

A shows WWTP-A; B shows WWTP-B.

**Table 3:** Sequence of primers used for detecting *cpe.*

Primer	Sequence (5′–3′)	Product Size (bp)
cpe-F	GATAAAGGAGATGGTTGGATATTAGGGGAAC	383
cpe-R	CCTAAGCTATCTGCAGATGTTTTACTAAGCC	

**Table 4:** Characteristics of type F *C. perfringens* assessed by MLST.

Strain	Location of *cpe*	*cpb2*	Source	Isolation Date
14-12	Plasmid with an IS*1151* sequence	+	Human feces in non-foodborne case	June 2014
14-14	Plasmid with an IS*1151* sequence	+	Human feces in non-foodborne case	July 2014
18-229	Plasmid with an IS*1151* sequence	+	Human feces in non-foodborne case	November 2018
14-15	Plasmid with an IS*1151* sequence	+	Human feces in non-foodborne case	July 2014
14-133	Plasmid with an IS*1151* sequence	+	Human feces in non-foodborne case	December 2014
19-14	Plasmid with an IS*1151* sequence	+	Human feces in non-foodborne case	April 2019
16-101	Plasmid with an IS*1151* sequence	–	Sewage influent from WWTP-A	July 2016
17-29	Plasmid with an IS*1151* sequence	–	Sewage influent from WWTP-A	October 2016
17-82	Plasmid with an IS*1151* sequence	+	Sewage influent from WWTP-A	December 2016
18-101	Plasmid with an IS*1151* sequence	–	Sewage influent from WWTP-A	January 2018
19-100	Plasmid with an IS*1151* sequence	+	Sewage influent from WWTP-A	June 2019
17-37	Plasmid with an IS*1151* sequence	+	Sewage influent from WWTP-B	November 2016
17-100	Plasmid with an IS*1151* sequence	+	Sewage influent from WWTP-B	December 2016
18-29	Plasmid with an IS*1151* sequence	+	Sewage influent from WWTP-B	July 2017
18-340	Plasmid with an IS*1151* sequence	+	Sewage influent from WWTP-B	November 2018
19-43	Plasmid with an IS*1151* sequence	+	Sewage influent from WWTP-B	March 2019
18-350	Plasmid with an IS*1151* sequence	+	Sewage effluent from WWTP-A	February 2019
19-16	Plasmid with an IS*1151* sequence	–	Sewage effluent from WWTP-A	March 2019
18-353	Plasmid with an IS*1151* sequence	+	Sewage effluent from WWTP-B	January 2019
19-5	Plasmid with an IS*1151* sequence	–	Sewage effluent from WWTP-B	February 2019
19-124	Plasmid with an IS*1151* sequence	+	Asari clam from Aichi	July 2019
19-141	Plasmid with an IS*1151* sequence	+	Asari clam from Aichi	August 2019
19-146	Plasmid with an IS*1151* sequence	+	Asari clam from Aichi	September 2019
19-169	Plasmid with an IS*1151* sequence	–	Asari clam from Kumamoto	November 2019
19-170	Plasmid with an IS*1151* sequence	+	Asari clam from Kumamoto	November 2019
19-171	Plasmid with an IS*1151* sequence	+	Asari clam from Kumamoto	December 2019
19-172	Plasmid with an IS*1151* sequence	+	Asari clam from Kumamoto	December 2019
14-13	Plasmid with an IS*1470*-like sequence	+	Human feces in non-foodborne case	June 2014
13-11	Plasmid with an IS*1470*-like sequence	–	Human feces in non-foodborne case	April 2013
19-15	Plasmid with an IS*1470*-like sequence	+	Human feces in non-foodborne case	April 2019
18-25	Plasmid with an IS*1470*-like sequence	+	Sewage influent from WWTP-A	July 2017
17-36	Plasmid with an IS*1470*-like sequence	–	Sewage influent from WWTP-B	January 2016
18-268	Plasmid with an IS*1470*-like sequence	–	Sewage influent from WWTP-B	August 2018
18-262	Plasmid with an IS*1470*-like sequence	–	Sewage effluent from WWTP-B	August 2018
18-289	Plasmid with an IS*1470*-like sequence	–	Sewage effluent from WWTP-B	December 2018
19-62	Plasmid with an IS*1470*-like sequence	–	Sewage effluent from WWTP-B	April 2019
